# Supplementary material for: The Oral Inactivated Porcine Epidemic Diarrhea Virus Presenting in the Intestine Induces Mucosal Immunity in Mice with Alginate–Chitosan Microcapsules
Source: Animals (Basel). 2023 Feb 28;13(5):889. doi: 10.3390/ani13050889 (PMC10000104; doi:10.3390/ani13050889)
Supplement: Supplementary file 1 [file animals-13-00889-s001.zip › animals-2104007-supplementary.pdf]

Table S1. Primers used for q-RT PCR for inflammatory and functional analyses.

Primer	Forward sequence	Reverse sequence
β-actin	CCAGTTGGTAACAATGCCATGT	GGCTGTATTCCCCTCCATCG
IL-4	GTCATCCTGCTCTTCTTTCTCG	ATGGCGTCCCTTCTCCTGT
IL-1β	AGCTTCAAATCTCGCAGCAG	TCTCCACAGCCACAATGAGT
IL-10	CGCAGCTCTAGGAGCATGTG	GCTCTTACTGACTGGCATGAG
TNF-α	AGTGGTGCCAGCCGATGGGTTGT	GCTGAGTTGGTCCCCCTTCTCCAG
IFN-γ	AGACAATCAGGCCATCAGCA-	AGACAATCAGGCCATCAGCA
IL-17	GCTCCAGAAGGCCCTCAGACTACC	TTCCCTCCGCATTGACACAGC
TGF-β	GAGAAGAACTGCTGTGTGCG	GTGTCCAGGCTCCAAATATAG
occlutin	GCTGTGATGTGTGTGAGCTG	GACGGTCTACCTGGAGGAAC
ZO-1	AGCGAAAACCCGAAACTGATG	TGATACTGAGTTGCCTTCACCCT
